# PROMs reveal early fall risk in the absence of clinical disability in people with multiple sclerosis

**DOI:** 10.1007/s10072-025-08605-w

**Published:** 2026-01-07

**Authors:** Nurşen Kurtuluş, Aysun Ünal, Bengü Altunan, Filiz Dilek, Aslı Aksoy Gündoğdu, Birol Topçu

**Affiliations:** 1 Faculty of Medicine, Department of Neurology, Tekirdağ Namık Kemal University, Tekirdağ, Turkey; 2 Vocational School of Health Services, Elderly Care Program, Tekirdağ Namık Kemal University, Tekirdağ, Turkey; 3 Faculty of Medicine, Department of Biostatistics, Tekirdağ Namık Kemal University, Tekirdağ, Turkey

**Keywords:** Multiple sclerosis, Falls, Fear of falling, PROMs, MSWS-12, Balance

## Abstract

**Background:**

Falls and fear of falling (FoF) are common in people with multiple sclerosis (pwMS), even in the absence of significant physical disability. To identify the determinants of falls in pwMS with low disability (EDSS < 4.0) and to examine the utility of patient-reported outcome measures (PROMs) in guiding timely fall prevention strategies.

**Methods:**

Fifty RRMS patients (EDSS < 4.0, aged 18–50 years) and 30 age, sex, and education-matched healthy controls were assessed. Participants were evaluated for fall history (retrospective and prospective), static and dynamic balance, gait speed (T25FW), walking limitations (MSWS-12), cognitive function (MoCA), depression (BDI-II), and fear of falling (FES-I). Correlation and regression analyses were conducted to identify factors associated with FoF.

**Results:**

Falls occurred in 48% of pwMS and 30% of healthy subjects (HC) (*p* = 0.1). FoF was significantly more common in pwMS (50%) than in HCs (23.3%, *p* = 0.001). MS patients who fell had significantly higher MSWS-12 and FES-I scores than non-fallers (*p* = 0.001 and *p* = 0.01, respectively). FoF positively correlated with age, disease duration, EDSS, static balance, and MSWS-12, and negatively with education level. In linear regression, MSWS-12 (*p* < 0.001) and disease duration (*p* = 0.03) were significant predictors of FoF.

**Conclusion:**

FoF is common among pwMS even at low disability levels and may contribute to fall risk and activity restriction. PROMs, particularly MSWS-12 and FES-I, are valuable tools for identifying fall-prone individuals and should be integrated into routine clinical assessments to enable early intervention.

**Supplementary Information:**

The online version contains supplementary material available at 10.1007/s10072-025-08605-w.

## Introduction

Multiple sclerosis (MS) is a chronic, inflammatory, immune-mediated neurodegenerative disease of the central nervous system, characterized by demyelination and varying degrees of axonal damage [1]. Depending on which neurological systems are affected and when, patients may experience a range of symptoms including fatigue, neuropsychiatric disturbances, motor, sensory, visual, cerebellar, and brainstem dysfunctions. Impairments in muscle strength, sensation, and balance contribute to postural instability and, consequently, increase the risk of falls. According to the literature, falls occur in approximately 54%–56% of people with MS (pwMS) [2]. Physiological factors linked to falls include higher disability levels, progressive disease course, assistive device usage, and impaired balance [3]. Notably, a consistent correlation between fall status and the Expanded Disability Status Scale (EDSS) has been observed only at intermediate EDSS levels (4.0–6.0) [4, 5]. Most studies investigating falls in pwMS report mean EDSS scores greater than 3.0 [6–8]. However, even in the absence of overt clinical disability, pwMS can experience subclinical impairments in balance, gait, and cognitive function. There remains limited research exploring the prevalence and determinants of falls in this subgroup of non-disabled individual [9, 10].

Falls may also lead to the development of fear of falling (FoF), which can result in reduced physical activity, deconditioning, and decreased independence [5]. Importantly, FoF has been reported not only among those with a history of falls but also in patients who have never experienced a fall [11, 12]. Awareness of potential fall-related consequences may lead to anticipatory anxiety and diminished confidence in performing daily tasks [13]. Kalron and Allali (2017) found no significant differences in gait parameters between fearless fallers and non-fallers, but patients with FoF and a history of falls exhibited greater neurological impairment, slower walking speed, reduced stride length, and greater bilateral gait asymmetry. Interestingly, patients with FoF but no falls also differed in gait characteristics compared to fearless fallers [14]. These findings suggest that gait disturbances in pwMS may be strongly influenced by FoF, highlighting the need for behavioral approaches targeting FoF in fall prevention strategies.

Falls are frequently underreported and underrecognized in clinical practice. Implementing quick, easy screening tools in outpatient settings could facilitate the early identification of at-risk patients and allow timely referrals to rehabilitation services. It is also essential to assess whether patients experience FoF. Identifying fallers and the associated risk factors before the onset of clinical disability may inform early therapeutic interventions. Patient-reported outcome measures (PROMs), which capture patients’ subjective assessments of their health status, are increasingly used in clinical practice [15]. However, there is still limited evidence regarding the use of PROMs in fall risk assessment and management in MS outpatient care.

Therefore, the aim of this study was to identify the determinants of falls in pwMS who do not require intermittent or unilateral support for daily activities, and to evaluate the utility of PROMs in guiding timely fall prevention interventions.

## Methods

A total of 68 patients with relapsing-remitting MS (RRMS), aged between 18 and 50 years, with an EDSS score of less than 4.0 and no history of relapse within the past 60 days, were evaluated. Participants were consecutively recruited from the outpatient MS clinic of the Neurology Department. Patients were excluded if they had orthopedic or visual impairments that could adversely affect mobility, were pregnant, were taking 4-Aminopyridine, or had concurrent chronic systemic diseases or depression. Additionally, 30 healthy volunteers without chronic illnesses, matched to the patient group based on age, sex, and educational status, were recruited as the control group.

Participants were interviewed regarding their fall history in the previous year, and the number of falls was recorded. Fall status was also assessed prospectively during the 6-month follow-up period via monthly face-to-face or telephone interviews. Each participant was evaluated in two separate sessions: balance and walking parameters were assessed in one session, and neuropsychiatric parameters were evaluated in the other.

Eighteen participants withdrew from the study voluntarily during follow-up. The final analysis included data from 50 RRMS patients and 30 healthy controls, with no missing data.

### Data collection instruments

#### Questionnaire form

Developed by the research team, this questionnaire included items on age, sex, disease duration (years), and education level (years). It also tracked fall history, including the number of falls in the past year and monthly follow-up over a six-month period. A fall was defined as an unexpected event in which the participant comes to rest on the ground, floor, or a lower level. PwMS who reported one or more falls either retrospectively or prospectively were classified as “fallers” [16].

#### Expanded Disability Status Scale (EDSS)

The EDSS is a widely accepted tool that evaluates disability status ranging from 0 (no disability) to 10 (death), based on neurological examination and walking ability [17]. EDSS scores between 1.0 and 4.0 indicate that patients do not require intermittent or unilateral support for daily activities. Assessments were conducted by a senior MS neurologist.

#### Timed 25-Foot walk test (T25FW)

This test measures the time required to walk 7.62 m and is commonly used to evaluate mobility and lower extremity function in PwMS. Patients are instructed to walk as quickly and safely as possible in both directions. The average of two trials is calculated as the final score [18].

#### Balance tests

All balance assessments were performed using the Korebalance™ device at the NKU Health Research and Training Hospital, Physical Therapy and Rehabilitation Unit. The system includes a pneumatic mobile platform with side handles and a front display. During the static test, participants attempt to maintain a cursor in the center of a screen for 30 s. For the dynamic test, participants follow a moving red ball on the screen for 30 s, once with hands on the handles and once without. As per the device manual, scores above 700 for static balance and above 2000 for dynamic balance are considered risky. Higher scores indicate poorer balance.

#### Beck Depression Inventory-II (BDI-II)

BDI-II is a 21-item self-report inventory used to assess the severity of depressive symptoms. Each item is rated from 0 to 3, yielding a total score ranging from 0 to 63. Scores ≥ 17 indicate clinical depression and a need for medical treatment. Patients with scores ≥ 17 were excluded from the study [19, 20].

#### Montreal Cognitive Assessment (MoCA)

MoCA is a brief screening tool used to assess cognitive domains such as attention, executive function, memory, language, visuospatial ability, abstract thinking, calculation, and orientation. It is validated for use in PwMS [21]. The Turkish version has a cut-off score of 21 for cognitive impairment [22].

#### Multiple Sclerosis Walking Scale (MSWS-12)

MSWS-12 is a patient-reported outcome measure assessing walking difficulties over the past two weeks. It includes 12 items scored from 1 to 3 (first 3 items) and 1–5 (remaining 9 items). The total score is transformed into a percentage scale. Higher scores indicate more severe walking limitations [23, 24].

#### Falls Efficacy Scale – International (FES-I)

FES-I is a valid and reliable instrument for evaluating fear of falling in PwMS. It consists of 16 items rated from 1 (“not at all concerned”) to 4 (“very concerned”) for various daily activities. A cut-off score of 24 was used to distinguish patients with and without FoF [25, 26].

### Statistical analysis

Data were analyzed using PASW Statistics 18 for Windows. Descriptive statistics (mean, median ± SD, min–max, frequencies) were used to describe participant demographics and clinical characteristics. The Kolmogorov-Smirnov test was used to assess normality. Between-group comparisons were conducted using the independent samples t-test (for normally distributed data) and the Mann-Whitney U test (for non-normally distributed data). Spearman’s correlation coefficient was used to examine relationships between variables. The Chi-square test was applied to compare categorical variables. Linear regression analysis was used to evaluate the factors affecting FoF. Statistical significance was set at *p* < 0.05.

Following completion of the study, an observed effect size and post-hoc power analysis were conducted for the primary outcome measure of fear of falling (FES-I). The FES-I scores in the MS group (*n* = 50) and the control group (*n* = 30) yielded an observed Cohen’s d of 0.758. This effect size was entered into G*Power 3.1 using the “t tests – Means: Difference between two independent means (two groups)” procedure with a two-tailed α of 0.05. The resulting post-hoc power (1–β) was approximately 0.90, indicating that the achieved sample size was sufficient to detect a statistically significant difference in FES-I scores between the groups. For all key outcomes, effect sizes and 95% confidence intervals (CI) were reported in addition to p-values.

## Results

### Demographic characteristics of MS patients and HCs

The mean age of the 50 RRMS patients was 36.7 years, while the mean age of the 30 HCs was 36.6 years. The duration of disease ranged from 1 to 26 years; 50% of the patients had been diagnosed for less than 5 years, and 77% for less than 10 years. EDSS scores ranged from 1.0 to 4.0, with a median score of 2.0. Among the MS patients, 58% had an EDSS score of ≤ 2.0, and 84% had a score of ≤ 3.0. No significant differences were found between the patient and control groups in terms of age, sex, or educational status (Table 1). A comparison between participants who completed the study (*n* = 50) and those who withdrew (*n* = 18) revealed no significant baseline differences in age, sex, disease duration, disability level, MSWS-12, or FES-I (all *p* > 0.05). Therefore, attrition was not considered to have introduced systematic bias into the study findings.

**Table 1:** The demographic and clinical characteristics of the PwMS and HCs

Variable	RRMS (*n* = 50) Mean ± SD / *n*	HCs (*n* = 30) Mean ± SD / *n*	HL Median Difference	95% CI	*p*-value
Age, year	36.7 ± 8.2	36.6 ± 8.3	0	(-4)-4	0.9
Gender, F/M	43 / 7	26/4			0.9
Education, year	8.5 ± 3.9	8.8 ± 4.0	0	(-1)-0	0.7
Disease duration, year	7.4 ± 6.5	–	–	–	–
EDSS	2.5 ± 1.1	–	–	–	–
Static balance	500.2 ± 309.7	356.0 ± 157.5	95	(-6)-201	0.07
Dynamic balance (hands on)	1369.6 ± 617.0	1277.6 ± 522.4	72	(-168)-307	0.5
Dynamic balance (hands off)	1889.5 ± 875.4	1645.3 ± 640.1	179	(-83)-486	0.2
FES-I	26.0 ± 8.7	20.5 ± 3.7	4	1–6	0.001*
MoCA	22.0 ± 4.4	23.1 ± 3.6	(-1)	(-3)-1	0.3
BDI	12.2 ± 9.4	8.8 ± 5.9	2	(-1)-6	0.2
T25-FW (sec)	14.6 ± 6.7	–	–	–	–
MSWS-12	28.0 ± 29.2	–	–	–	–

Hodges–Lehmann estimators indicate median differences between the MS and HC groups, with corresponding 95% confidence intervals and p-values derived from the Mann–Whitney U tests

*p < 0.05 was considered statistically significant

### Comparison between PwMS and HC groups & fall risk evaluation

Among the 50 RRMS patients, a total of 48 falls were recorded, affecting 24 individuals (48%). Retrospectively, 16 falls (22%) were reported within the previous year, and 32 falls (34%) occurred during the six-month follow-up period. Nine participants (18%) were classified as recurrent fallers. In the HC group, 14 falls were reported by 9 individuals (30%), with three classified as recurrent fallers (Table 2).

Although the odds ratio (OR) for falling in the MS group was 2.2, this did not reach statistical significance (*p* = 0.1). Similarly, there was no significant difference between groups regarding recurrent falls (OR = 1.2, *p* = 0.8).

According to the static balance cut-off values, 22% of RRMS patients and 3.3% of controls were considered at risk. However, the difference in static balance scores between groups was not statistically significant (*p* = 0.07). Risky scores were observed in 14% (hands-on) and 36% (hands-off) of RRMS patients in the dynamic balance tests, compared to 6.7% and 20% of HCs, respectively. These differences were also not statistically significant (*p* = 0.497 and *p* = 0.188).

FoF, as measured by FES-I, was present in 50% of RRMS patients and 23.3% of controls, with MS patients showing significantly higher levels (*p* = 0.001). No statistically significant differences were found between pwMS and HCs in MoCA or BDI-II scores (*p* = 0.3 and *p* = 0.2, respectively).

**Table 2:** Number of individuals with falls and recurrent falls in the MS and HC groups

Group	Period	Recurrent fallers	Fallers	Total
MS group (*n* = 50)	Retrospective	1	6	7
	Prospective	4	9	13
	Retrospective and prospective	4	0	4
	Total	9	15	24
HC group (*n* = 30)	Retrospective	3	0	3
	Prospective	0	6	6
	Total	3	6	9

Fallers refer to individuals who experienced ≥ 1 fall; recurrent fallers refer to individuals who experienced ≥ 2 falls in the specified period

### Comparison between PwMS with and without falls

Of the 50 MS patients, 24 reported falls. Compared to non-fallers, fallers had significantly higher MSWS-12 and FES-I scores (*p* = 0.001 and *p* = 0.01, respectively). No significant differences were found between in age, gender, education, disease duration, EDSS, static/dynamic balance scores, MoCA, BDI-II, or T25-FW performance (Table 3).

**Table 3:** Comparison of demographic and clinical variables between MS patients with falls (MSwF) and without falls (MSwoF)

Variable	MSwF (*n* = 24) Mean ± SD / *n*	MSwoF (*n* = 26) Mean ± SD / *n*	HL Median Difference	95% CI	*p*-value
Age, year	38.3 ± 7.6	35.3 ± 8.6	(-3)	(-8)-2	0.2
Gender, F/M	22/2	21/5			0.3
Education, year	8.0 ± 3.8	9.0 ± 4.0	0	0–3	0.4
Disease duration, year	7.7 ± 7.2	7.1 ± 5.9	0	(-3)-3	0.9
EDSS	2.7 ± 1.2	2.2 ± 1.0	(-0.3)	(-1)-0	0.1
Static balance	571.3 ± 316.3	434.5 ± 294.4	(-129)	(-286)-9	0.07
Dynamic balance (hands on)	1311.0 ± 455.2	1423.8 ± 741.0	32	(-259)-404	0.9
Dynamic balance (hands off)	1850.1 ± 593.1	1925.9 ± 1084.2	(-119.5)	(-476)-378	0.7
FES-I	29.4 ± 10.0	22.9 ± 6.0	(-5)	(-11)-(-1)	0.01*
MoCA	21.0 ± 4.0	22.8 ± 4.7	2	(-1)-4	0.1
BDI	13.7 ± 9.3	10.9 ± 9.4	(-3)	(-8)-2	0.2
T25-FW (sec)	13.8 ± 5.8	15.3 ± 7.5	0	(-1)-3	0.8
MSWS-12	41.4 ± 30.1	15.6 ± 22.6	(-28)	(-43)-(-7)	0.001*

Hodges–Lehmann estimators indicate median differences between the MSwF and MSwoF groups, with corresponding 95% confidence intervals and p-values derived from the Mann–Whitney U tests

*p < 0.05 was considered statistically significant

### Correlation between FoF and other factors

FoF was positively correlated with age (*r* = 0.285, *p* < 0.05), EDSS (*r* = 0.443, *p* < 0.001), disease duration (*r* = 0.292, *p* < 0.05), static balance (*r* = 0.312, *p* < 0.05), and MSWS-12 scores (*r* = 0.700, *p* < 0.001). A negative correlation was found between FoF and education level (*r* = -0.331, *p* < 0.05) (Table 4).

**Table 4:** Correlation matrix of variables associated with fear of falling (FES-I) in patients with MS

Variable	FES-I	Age	Education	EDSS	Disease duration	Static balance	Dynamic balance (hands on)	Dynamic balance (hands off)	T25-FW	MSWS-12
FES-I	1.000	0.285*	–0.331*	0.443**	0.292*	0.312*	0.276	0.195	0.211	0.700**
Age	0.285*	1.000	–0.527**	0.257	0.332*	0.357*	0.341*	0.244	0.006	0.387**
Education	–0.331*	–0.527**	1.000	–0.233	–0.082	–0.181	–0.437**	–0.301*	–0.139	–0.381**
EDSS	0.443**	0.257	–0.233	1.000	0.464**	0.461**	0.170	0.263	0.161	0.621**
Disease duration	0.292*	0.332*	–0.082	0.464**	1.000	0.342*	0.224	0.275	–0.066	0.346*
Static balance	0.312*	0.357*	–0.181	0.461**	0.342*	1.000	0.441**	0.520**	–0.125	0.420**
Dynamic balance (hands on)	0.276	0.341*	–0.437**	0.170	0.224	0.441**	1.000	0.765**	0.045	0.315*
Dynamic balance (hands off)	0.195	0.244	–0.301*	0.263	0.275	0.520**	0.765**	1.000	–0.111	0.341*
T25-FW	0.211	0.006	–0.139	0.161	–0.066	–0.125	0.045	–0.111	1.000	0.065
MSWS-12	0.700**	0.387**	–0.381**	0.621**	0.346*	0.420**	0.315*	0.341*	0.065	1.000

Correlation coefficients are based on Spearman’s r

Statistical significance: *p < 0.05, **p < 0.01

### Linear regression analysis of predictors of FoF

Variables included in the linear regression model were age, education, disease duration, EDSS, static balance, dynamic balance, T25-FW, and MSWS-12. Among these, MSWS-12 (*p* < 0.001) and disease duration (*p* = 0.03) emerged as significant predictors of FoF. Specifically, each one-point increase in MSWS-12 was associated with a 0.221-point increase in the FES-I score, and each additional year of disease duration was associated with a 0.379-point increase. Other variables did not significantly predict FoF in the model (Table 5).

**Table 5:** Linear regression analysis of factors associated with fear of falling (FES-I) in MS patients

Predictor	B (95% CI)	Std. Error	β	t	*p*	Zero-order	Partial
(Constant)	30.438 (15.201–45.675)	7.539	–	4.037	0.000	–	–
Age	–0.178 (–0.463–0.108)	0.141	–0.167	–1.256	0.216	0.285	–0.195
Education	–0.409 (–1.008–0.189)	0.296	–0.184	–1.384	0.174	–0.331	–0.214
EDSS	–1.703 (–3.972–0.567)	1.123	–0.221	–1.516	0.137	0.378	–0.233
Disease duration	0.379 (0.046–0.711)	0.165	0.282	2.301	0.027*	0.346	0.342
Static balance	0.001 (–0.007–0.009)	0.004	0.037	0.266	0.792	0.350	0.042
Dynamic balance (hands-on)	0.003 (–0.003–0.008)	0.003	0.196	1.040	0.304	0.229	0.162
Dynamic balance (hands-off)	–0.002 (–0.005–0.002)	0.002	–0.193	–1.103	0.277	0.102	–0.172
MSWS-12	0.221 (0.137–0.306)	0.042	0.741	5.288	0.000*	0.696	0.641
T25-FW	0.007 (–0.277–0.292)	0.141	0.006	0.053	0.958	0.103	0.008

Model summary: F = 6.168, *p* < 0.001, Adjusted R² = 0.487, Standard error = 6.253 *p < 0.05 was considered statistically significant

## Discussion

This study aimed to evaluate whether falls in pwMS who do not require support for daily activities can be predicted using PROMs. Although the overall frequency of falls did not differ significantly between pwMS and HCs, pwMS reported significantly greater FoF. FoF was also significantly more common among those who had experienced at least one fall.

FoF is widely recognized as a psychological response to actual or perceived fall risk and is linked to decreased confidence in physical and social functioning. Prior studies have shown that FoF can lead to substantial activity restriction even in the absence of previous falls, with up to 82.6% of pwMS avoiding activity due to FoF [27]. In this context, the absence of a statistically significant difference in fall rates between pwMS and controls in the present study may be explained by behavioral adaptations - such as reduced physical engagement - driven by elevated FoF. While lower extremity muscle weakness is often cited as a cause of FoF, our findings support the alternative hypothesis that FoF and resulting activity limitation may themselves contribute to deconditioning over time [5, 11].

Our data showed that MSWS-12 scores, reflecting self-perceived walking impairment, were significantly higher in both fallers and pwMS with FoF. Notably, 81.8% of participants with MSWS-12 scores exceeding 50 reported at least one fall during the study period, supporting a strong link between greater subjective gait limitation and increased fall risk. MSWS-12 also showed significant positive correlations with objective mobility metrics such as T25-FW and static balance performance. These findings reinforce the utility of the MSWS-12 as a sensitive PROM for capturing not only functional mobility but also subtle impairments in gait confidence and postural control [28, 29]. Importantly, MSWS-12 and disease duration were identified as independent predictors of FoF in the regression model. This supports the view that PROMs can capture complex functional and psychological dimensions of fall risk, which may not be detectable through conventional clinical tests. While T25-FW is an objective measure of gait speed, it may not fully reflect coordination, fatigue, or anticipatory postural responses. In contrast, the MSWS-12 appears to encompass these broader experiential aspects. Additionally, these findings align with recent literature suggesting that elevated FoF may serve as an early behavioral marker of compromised motor control - both internal (e.g., proprioception) and external (e.g., navigating complex environments) - even before observable clinical deficits emerge [5, 30]. This suggests that higher MSWS-12 scores in fallers may not merely indicate physical limitations, but also a heightened awareness or anticipation of instability, even in the absence of objective gait abnormalities. These insights underscore the importance of integrating PROMs into clinical practice, especially in early-stage MS patients.

Self-assessment tools such as MSWS-12 may provide critical information regarding patients’ lived experiences of mobility limitations, compensatory behaviors, and psychological responses to subtle motor dysfunction. Early identification of such signals could prompt timely interventions aimed at mobility training, balance enhancement, or cognitive-behavioral strategies to reduce FoF and prevent avoidable activity restriction or future falls. PROMs provide unique insight into patients’ perceived mobility, confidence, and behavioral adaptations, which may not be captured through performance-based tests alone. In early MS, subtle gait or balance disturbances may not be detectable on clinical examination, whereas PROMs such as MSWS-12 or FES-I can identify emerging concerns that reflect real-world functioning. Integrating PROMs with objective assessments may therefore enhance early detection of mobility decline and support more personalized fall-prevention strategies.

Regarding balance, RRMS participants demonstrated poorer static balance performance than controls, with a higher proportion falling into the “at-risk” category based on cut-off scores. Among pwMS, those with FoF and a history of falls exhibited particularly worse balance scores. This finding supports prior research showing that balance impairments may be detectable even in pwMS with low EDSS scores [9, 31]. Kalron and Achiron (2013) similarly reported significant associations between FoF and balance metrics [2] Our data thus suggest that static balance may be a sensitive physical correlate of FoF and that self-report tools like MSWS-12 or FES-I may reflect these changes before they manifest clinically.

Several assessment methods exist to evaluate walking and fall risk in pwMS, including laboratory-based gait analysis, performance-based tests such as T25-FW, and PROMs. While laboratory methods are highly accurate, they are resource-intensive and less feasible in routine care [32]. T25-FW provides objective, rapid assessment but may fail to capture subjective or contextual mobility challenges [33]. PROMs such as MSWS-12 and FES-I offer accessible, time-efficient alternatives that reflect real-world experiences and psychological dimensions of fall risk [23, 25, 34]. Our results support the combined use of MSWS-12 and FES-I in identifying fall risk in ambulatory pwMS with low disability, highlighting their value in clinical decision-making and early intervention planning.

### Limitations

This study has several limitations. The relatively small sample size may limit the generalizability of the findings. While MoCA was used as a practical cognitive screening tool, more detailed neuropsychological testing could provide deeper insights into the cognitive contributions to FoF. That said, excluding participants with depression or significant cognitive impairment allowed for a more focused assessment of the relationship between FoF and functional status.

## Conclusions

In conclusion, this study highlights the clinical importance of assessing FoF even in pwMS with minimal disability. Self-reported measures such as MSWS-12 and FES-I provide valuable insight into early mobility concerns, psychological adaptations, and fall-related risk. PROMs should be routinely integrated into clinical practice to facilitate timely, patient-centered interventions aimed at reducing fall risk and improving quality of life.

## Supplementary Information

Below is the link to the electronic supplementary material.

Supplementary Material 1
